# Boron‐Locked Starazine – A Soluble and Fluorescent Analogue of Starphene

**DOI:** 10.1002/chem.202200770

**Published:** 2022-04-07

**Authors:** Yi Feng, Jiadong Zhou, Honglin Qiu, Matthias Schnitzlein, Jingtao Hu, Linlin Liu, Frank Würthner, Zengqi Xie

**Affiliations:** ^1^ State Key Laboratory of Luminescent Materials and Devices Institute of Polymer Optoelectronic Materials and Devices Guangdong Provincial Key Laboratory of Luminescence from Molecular Aggregates South China University of Technology (SCUT) 510640 Guangzhou P. R. China; ^2^ Institut für Organische Chemie & Center for Nanosystems Chemistry Universität Würzburg Am Hubland 97074 Würzburg Germany

**Keywords:** conjugated molecule, electronic structure, luminescence, starazine, starphene analogue

## Abstract

A starlike heterocyclic molecule containing an electron‐deficient nonaaza‐core structure and three peripheral isoquinolines locked by three tetracoordinate borons, namely isoquinoline‐nona‐starazine (QNSA), is synthesized by using readily available reactants through a rather straightforward approach. This new heteroatom‐rich QNSA possesses a quasi‐planar π‐backbone structure, and bears phenyl substituents on borons which protrude on both sides of the π‐backbones endowing it with good solubility in common organic solvents. Contrasting to its starphene analogue, QNSA shows intense fluorescence with a quantum yield (PLQY) of up to 62 % in dilute solution.

## Graphical abstract

**The first counterpart** of starphene, namely starazine, that contains a nonaaza‐core structure and three peripheral isoquinoline units locked by three tetracoordinate borons to form a quasi‐planar backbone is described. This starazine possesses a non‐aromatic and electron‐deficient core due to the introduction of the heteroatoms replacing the corresponding carbon atoms in starphene. The transition to its lowest excited state is allowed, sharply contrasting to the starphenes, endowing fluorescent properties to this novel starlike dye molecule.

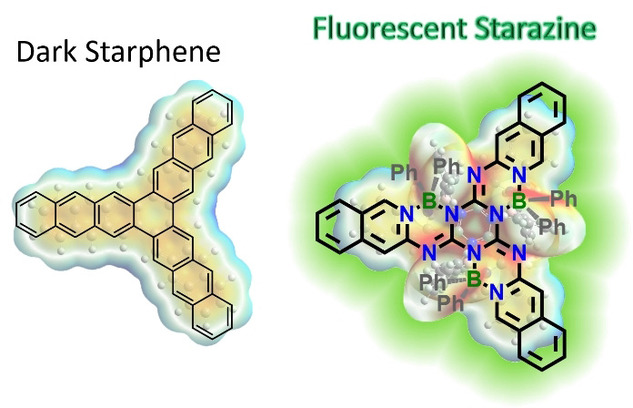

Starphenes^[1]^ are a class of polyaromatic hydrocarbons (PAHs) containing a central benzene ring with three extending fused acene arms (Figure 1 left shows an representative example). Starphenes are attractive conjugated molecules due to their unique electronic structure and potential applications in single‐molecule electronics and optoelectronic devices.^[2]^ However, the fusion of successive rings in the arms is challenging due to the low solubility in common solvents, and thus most of the reported starphene molecules were obtained by surface reactions, which greatly limits their synthesis and application as functional materials.^[3]^ In the past several years, Soe and Robles et al. successfully synthesized a set of aza‐starphenes and observed magnetic properties by anchoring various numbers of aluminium atoms in the bay area(s), which triggered the investigations of magnetic starphenes (Figure 1 middle).^[4]^ To the best of our knowledge, the reported starphenes are at best weakly fluorescent due to the symmetry‐forbidden transitions of the lowest excited state which limits their application in optoelectronics.

**Figure 1:**
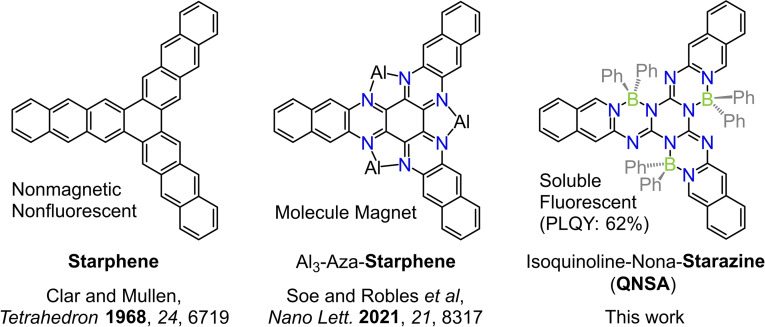
Chemical structures of previously reported representative starphene (left), magnetic Al_3_‐aza‐starphene (middle), and the structure of our fluorescent isoquinoline‐nona‐starazine (QNSA) (right).

During the last decade the replacement of sp^2^‐hybridized carbon in PAHs by heteroatoms afforded a plethora of successful molecular materials for organic electronics.^[5]^ Besides nitrogen,^[6]^ it was in particular the other neighbour element of carbon, i.e. boron that endowed the heteroatom‐doped PAHs with most useful functional properties.^[7]^ Indeed, there is an impressive variety how this element could be utilized including tricoordinate and tetracoordinate boron centers. One of the most fascinating replacements is provided by isoelectronic B←N as a substitute of C−C.^[8]^ However, despite of the resemblance of BN and CC units, either with formal single or double bond character, perturbations are severe. For instance, cyclic electron delocalization is not substantial anymore in borazine (B3N3H6) as the BN counterpart of benzene.^[9]^ Further, as most impressively exemplified by the highly successful emitter class of BODIPY dyes,^[10]^ configurational locking of aza‐heteroaromatic units by tetracoordinate boron afforded a broad variety of highly interesting fluorescent dyes.^[11]^ Accordingly, the field of novel organic emitters and luminescent materials has been strongly enriched by boron compounds. This has been recently augmented by very successful new blue TADF (thermally activated delayed fluorescence) emitters for OLEDs.^[12]^

Herein, we introduce a rather straightforward approach to synthesize a starlike heterocyclic π‐scaffold, which is proposed to be named as starazine (Figure 1 right). This starazine contains an electron‐deficient triazine core which is locked with three peripheral isoquinolines by three tetracoordinate borons, i.e. isoquinoline‐nona‐starazine (QNSA). This starazine molecule might be considered as the first counterpart of the family of starphenes, that contrastingly possesses a rather high photoluminescence quantum yield (PLQY) of 62 % in dichloromethane. QNSA has a rigid and quasi‐planar backbone structure due to the lock of the tetracoordinate boron atoms, while the phenyl substituents on borons protrude on both sides of the π‐backbones endowing it with good solubility in common solvents. In the following we reveal the structural and fluorescence properties of the first synthesized starphene analogue and thereby introduce a new class of tetracoordinate boron‐based functional dyes among other widely used conjugated small molecules like BODIPYs^[13]^ and polymers.^[14]^

The synthetic route towards our target compound, QNSA, is illustrated in Figure 2a. Firstly, following the established route for melamine derivatives,^[15]^ cyanuric chloride (**1**) was reacted with isoquinolin‐3‐amine (**2**) to give the triaminotriazine (**3**), which was collected by evaporating the reaction solvent and purifying the crude solid by thorough washing with ethanol. Then the intermediate triaminotriazine was reacted with triphenylborane to afford the starlike BN‐containing heterocyclic product QNSA through a three‐fold borylation reaction.^[16]^ Despite of the planarization of the QNSA π‐scaffold (for details see below) the solubility of QNSA is rather good in common solvents like toluene and dichloromethane due to the phenyl substituents that protrude above and below the π‐planes. Thus, the QNSA could be easily purified by column chromatography to afford a total yield of about 15 % for the two‐step reaction sequence. Considering that all the reactants and reagents are readily available, and also the reaction conditions are mild, this synthetic route should therefore be suitable to conveniently expand this new class of heteroatom‐rich starlike compounds. The chemical structure of QNSA was characterized by ^1^H NMR, ^13^C NMR, MS as well as single crystal X‐ray analysis. QNSA shows very good thermal stability, with decomposition temperature (*T*_d_) up to 416 °C as tested by thermogravimetric analysis (Figure S3).

**Figure 2:**
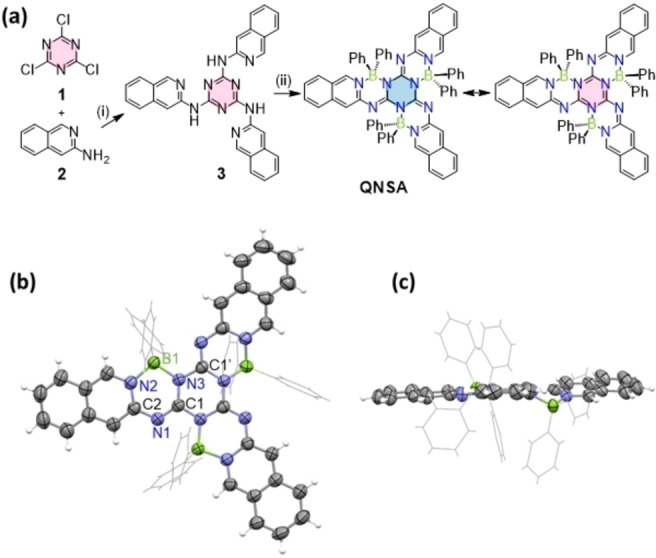
(a) Synthesis route for QNSA (with two resonance structures). Reaction condition: (i) Triethylamine, toluene, 110 °C, 12 h; (ii) triphenylborane, toluene, 110 °C, 48 h. (b) Top view of QNSA molecule in single crystal structure. Selective bond length (average value for the corresponding bonds in the three arms in one molecule): B1−N2, 1.610 Å; B1−N3, 1.616 Å; C1−N1, 1.311 Å; C2−N1, 1.354. (c) Side view of QNSA molecule.

Single crystals of QNSA were obtained by recrystallization from dichloromethane/ethanol mixture and the data from our single crystal X‐ray analysis are depicted in Table S1.^[17]^ As shown in Figure S6a, DFT calculations indicate that QNSA molecule is *C*_3_‐symmetric in vacuum, while the symmetry in the crystal is reduced which might be a packing effect. Whilst the heterocyclic backbone is close to planar (Figure 2b and 2c) and undoubtedly enables pronounced π‐conjugation along the whole π‐scaffold, we noted different distortions of the three arms of QNSA molecule in the single crystal. An almost perfectly planar π‐scaffold is established between two arms, thereby affording seven six‐membered rings in π‐conjugation, whereas the third arm is tilted out of the plane. As expected, due to the boron‐phenyl substituents protruding on both sides of the π‐backbones (Figure 2c), no cofacial π‐π‐stacking is possible for these molecules in the crystal (Figure S5). Nevertheless, slipped π‐π‐stacking arrangements involving the outer part of the π‐scaffolds, i.e. the isoquinoline units, can be observed in the crystal.

The B−N bonding in QNSA is worth being explored for its unique character in BN‐containing compounds. For some hexagonal rings containing the N−B←N unit, usually one shorter covalent bond (about 1.49 Å) and one longer coordination bond (about 1.58 Å) were found.^[18]^ However, in QNSA, the average bond length of B1‐N3 (the atom numbers are given in Figure 2b) is 1.616 Å, which is almost equal to that of B1‐N2 (1.610 Å). Thus, it is difficult to distinguish the covalent bond and coordination bond according to the B−N bond lengths (Table S2a). Therefore, the B−N bonding situation in the QNSA is rather comparable to that of phenyl‐substituted BODIPYs, in which B−N bonds were almost equal with the bond length of about 1.59 Å.^[19]^ Indeed, there are several possible resonance structures as shown in Figure S8. In order to determine the favored electronic structure, we examined the bond lengths of C1−N1 and C2−N1 in the crystal structures. The average bond length of C1‐N1 in QNSA is 1.311 Å, which is much shorter than that of C2−N1 bond (1.354 Å), indicating the double bonding for C1−N1 and single bonding for C2−N1. In addition, bond lengths from DFT calculations gave the same result (Table S2b). Accordingly, based on the double bond character of C1−N1, we assign B1−N2 as a coordinative bond and B1−N3 as a covalent bond. Hence, the backbone chemical structure of QNSA is best represented by the left structure shown in Figure 2a with aromatic isoquinoline units and a non‐aromatic triazine core (for details see below).

To elucidate the aromatic character of the triazine central unit and all hexagonal rings in these heterocyclic backbones we performed calculations of the anisotropy of the induced current‐density (AICD, see Figure 3a)^[20]^ and the nucleus independent chemical shifts (NICS, see Figure 3b).^[21]^ AICD plots show clockwise ring currents over the peripheral isoquinoline units, thereby confirming their aromatic character. In contrast, for the central triazine ring neither a clock‐ nor an anti‐clockwise ring current is induced, thereby suggesting the lack of aromatic or antiaromatic character. NICS calculations corroborate these results, showing strongly negative NICS(1)_ZZ_ values for isoquinoline rings (−20.41 and −21.31) and positive values for the central triazine ring (5.65) and the BN‐containing heterocycles (7.75). As a reference, the triazine ring in melamine shows a negative NICS(1)_ZZ_ value about −8.61 (Figure S9b), clearly exhibiting aromaticity. These results point out a weak anti‐aromatic character of the triazine core in QNSA, contrasting to the aromatic structure of the triazine units before borylation. Accordingly, the loss of aromaticity of the central triazine core is attributed to the introduction of the tetracoordinate boron locking units which change the electronic structure. Although the aromaticity is no longer maintained on the central triazine core and the BN‐containing heterocycles of the backbone, the π‐electrons delocalize on the whole backbone through the C1=N1−C2 bridges as shown in Figure S10. It should be noted that the tetracoordinate boron atoms do not contribute π‐electrons to the conjugation system. In addition, the central triazine ring reveals strong electronic negativity with blue colour in the electrostatic potential (ESP) map (Figure S11), displaying a strong polarization of the π‐surface, much different to the starphenes.

**Figure 3:**
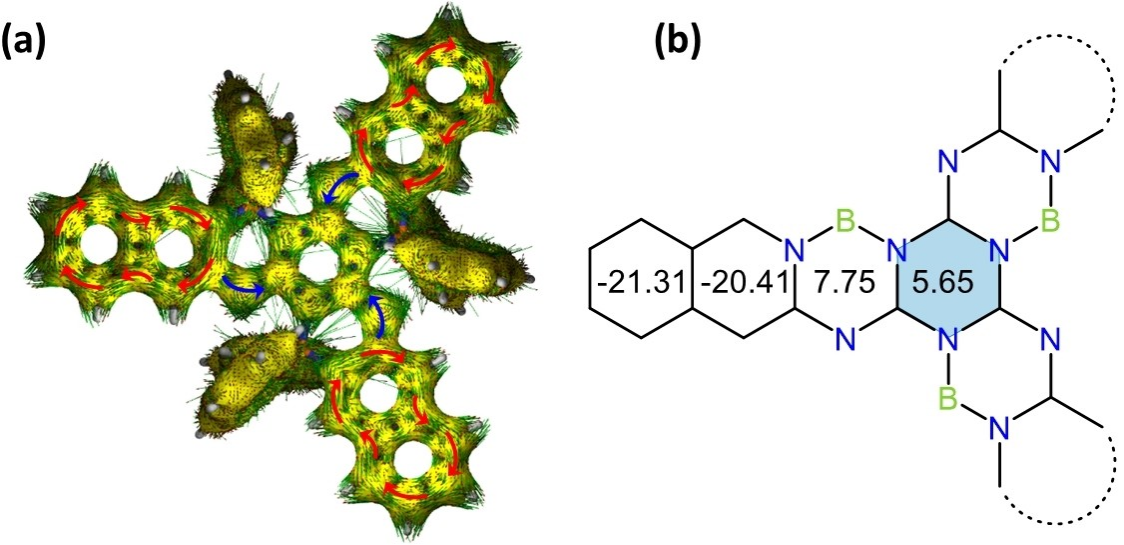
(a) Calculated AICD plots (isovalue: 0.05) of QNSA at B3LYP/6‐311+G(d,p) level of theory. Red arrows represent clockwise and blue arrows counter‐clockwise ring currents. (b) NICS(1)_zz_ of QNSA at B3LYP/6‐31+G(d) level of theory.

The absorption and fluorescence spectra of QNSA in dilute solution are shown in Figure 4a. Notably, QNSA exhibited a strong absorption band at ultraviolet region with maximum absorption peak at 358 nm and molar absorption coefficient of about 8.07×10^4^ L mol^−1^ cm^−1^, and a weaker absorption band at 390 ‐ 470 nm with molar absorption coefficient of about 2.67×10^4^ L mol^−1^ cm^−1^ (425 nm). Whichever excitation wavelength (425 or 358 nm) was selected, QNSA showed a broad and structureless emission. The photoluminescence quantum yield (PLQY) and fluorescence lifetime (*τ*) of QNSA in dilute dichloromethane solution were recorded as 62 % and 14 ns, which relates to radiative and non‐radiative rates of 0.043 and 0.027 ns^−1^, respectively. In addition, we changed the solvents for the spectroscopy measurements, and both the spectral shape and emission intensity (Figure S12) were observed to be insensitive to the polarity of the solvents, indicating that the emission was attributed to a locally excited (LE) transition.

**Figure 4:**
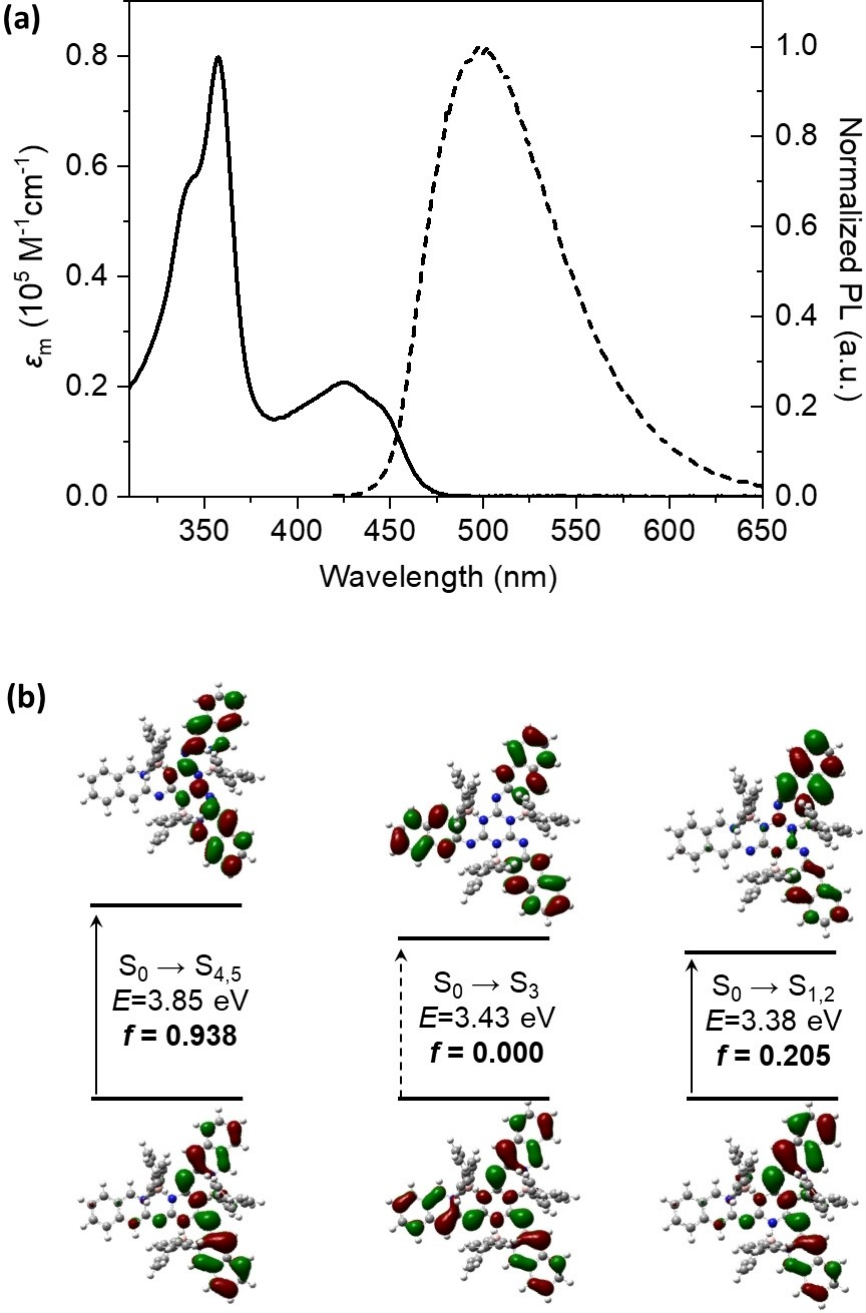
(a) Absorption (solid line) and photoluminescence (dashed line) spectra of QNSA in dilute dichloromethane solution (10^−5^ M). (b) NTOs of QNSA for the electronic transition from ground state to the excited states.

To understand the electronic excitations of QNSA, the frontier orbitals and natural transition orbitals (NTOs) were calculated at B3PW91/def2‐TZVP and M06‐2X/TZVP levels. As shown in Figures 4b and S13, there are expectant pairs of degenerate energy levels with equal energy (*E*) and oscillator strength (*f*), which exists in most *C*_3_‐symmetric molecules.^[22]^ QNSA exhibited a transition to the lowest degenerate levels (S_1,2_) with a smaller *f* of 0.205 and a larger electronic transition to the upper degenerate excited states (S_4,5_) with a larger *f* of 0.938, which matches well with the absorption spectra. Notably, for all allowed optical transitions of QNSA, the hole and particle were distributed on only two of the three arms of the backbone in the NTO pairs, which results in totally allowed transitions from the ground state to the degenerate excited states (S_1,2_ and S_4,5_); while the the hole and particle of S_3_ were distributed equally on three arms resulting in a symmetry‐forbidden transition (*f*=0). In contrast, for starphene the hole and particle for the lowest excited state (S_1_) delocalized at the whole conjugated plane (Table S3, Figure S14), and thus always suffers from low fluorescence.

In conclusion, a new starlike heterocyclic molecule, namely starazine, was designed and synthesized. As an analogue of starphene, the starazine contains a nonaaza‐core structure and three peripheral isoquinoline units which were locked by three tetracoordinate borons to form a quasi‐planar π‐scaffold. The introduction of BN units, replacing corresponding C atoms in starphene, not only reorganized the distribution of the internal π‐electrons of the backbone but also afforded a non‐aromatic core structure. NTOs calculation results showed that the hole and particle are located at only two of the three arms of the backbone, which enabled totally allowed transitions from the ground state to the lowest degenerate excited states. As a consequence, the QNSA π‐scaffold showed a structureless fluorescence spectrum with appreciable photoluminescence quantum yield of 62 % in dilute solution. The unusual electronic structure and fluorescence of the newly developed organic dye indicate the potential of starazines for both fundamental and application‐oriented investigations. Further experimental and theoretical studies are ongoing in our lab to reveal the features and functions of this class of heterocyclic starlike dyes.

## Conflict of interest

The authors declare no conflict of interest.

## Supporting information

As a service to our authors and readers, this journal provides supporting information supplied by the authors. Such materials are peer reviewed and may be re‐organized for online delivery, but are not copy‐edited or typeset. Technical support issues arising from supporting information (other than missing files) should be addressed to the authors.

Supporting Information

## Data Availability

The data that support the findings of this study are available from the corresponding author upon reasonable request.
