# Supplementary material for: Boron‐Locked Starazine – A Soluble and Fluorescent Analogue of Starphene
Source: Chemistry. 2022 Apr 7;28(29):e202200770. doi: 10.1002/chem.202200770 (PMC9325424; doi:10.1002/chem.202200770)
Supplement: Supplementary file 1 — Supporting Information [file CHEM-28-0-s001.pdf]

Chemistry–A European Journal

Supporting Information

Boron-Locked Starazine – A Soluble and Fluorescent Analogue of Starphene

Yi Feng, Jiadong Zhou,* Honglin Qiu, Matthias Schnitzlein, Jingtao Hu, Linlin Liu, Frank Würthner,* and Zengqi Xie*

Table of Contents

1. Materials and Methods S2

1.1 General S2

1.2 Spectroscopy S2

1.3 Thermo Gravimetric Analysis and Differential Scanning Calorimetry Measurements S2

1.4 X-Ray Diffraction S2

2. Synthesis and Characterization S3

3. Thermo Gravimetric Analysis and Differential Scanning Calorimetry S5

4. Single Crystal X-ray Analysis S6

5. Resonance Structures S9

6. Aromaticity S10

7. π-electron Analysis S11

8. Electrostatic Potential Map (ESP) S12

9. Absorption and Photoluminescence Spectra S13

10. Frontier Orbitals S14

11. Calculated Excited States and Spectra of Starphene S15

References S16

1. Materials and Methods

1.1 General

All reagents were purchased from commercial sources and used as received without further purification, unless otherwise stated. Triphenylborane (BPh_3) was prepared according to the reported procedures.[1] Reagent grade solvents were distilled prior to use. Column chromatography was performed on silica (silica gel, 300–400 mesh). ^{1}H, ^{13}C NMR spectra were recorded on a Bruker Avance III HD 400 spectrometer operating at 400 and 126 MHz. Chemical shifts were reported as δ values (ppm) relative to an internal tetramethylsilane (TMS) standard. *J* values are given in Hz. The following abbreviations were used to designate multiplicities: s = singlet, t = triplet, m = multiplet. High resolution mass spectra were obtained by matrix-assisted laser desorption/ionization (MALDI). MALDI spectra were recorded on Bruker Daltonics autoflex II LRF or Bruker Daltonics ultrafleXtreme spectrometers. *Trans*-2-[3-(4-tert-butylphenyl)-2-methyl-2-propenylidene]malononitrile (DCTB) or 2',4'-dihydroxyaceto-phenone (DHAP) were used as MALDI matrices.

1.2 Spectroscopy

UV/Vis spectra were recorded on a Shimadzu UV-3600 Plus spectrometer and the photoluminescence (PL) spectra were recorded by the Shimadzu RF-5301; all emission spectra were corrected for the wavelength sensitivity of the detection unit. All spectroscopy measurements were conducted with spectroscopic grade solvents from ACROS Organics. Conventional quartz cells (light path 1 cm) were used. The solvents used for spectra were toluene, o-dichlorobenzene (DCB), diethyl ether, tetrahydrofuran (THF) and dichloromethane (DCM). Unless otherwise noted, the solutions were measured at 10^{-5} M at room temperature.

Absolute quantum yields were determined by a calibrated integrating sphere (Hamamatsu Quantaurus-QY C11347-12). Fluorescence lifetimes were obtained by time-correlated single photon counting (TCSPC), using the Hamamatsu Quantaurus-Tau C11367-35 (365 and 425 nm laser excitation source). The lifetimes were fitted by exponentials through a deconvolution procedure with the instrumental response function (IRF).

1.3 Thermo Gravimetric Analysis and Differential Scanning Calorimetry Measurements

Thermo gravimetric analysis was tested by Netzsch Tg209f1, with heating range about 40 – 800 ℃ and heating rate about 20 ℃/min. Differential scanning calorimetry was tested by Netzsch DSC 200 F3 Maia, with heating/cooling range about 30 – 300 ℃ and heating/cooling rate about 20 ℃/min.

1.4 X-Ray Diffraction

Single crystal data for the QNSA were collected on Rigaku XtaLAB P2000 FR-X at 100 K. Single crystal structure was solved by ShelXT program using Intrinsic Phasing method and refined by ShelXL refinement package using Least Squares minimization, which worked on the Olex2 program. [2]

2. Synthesis and Characterization

Synthesis and Characterization of QNSA.

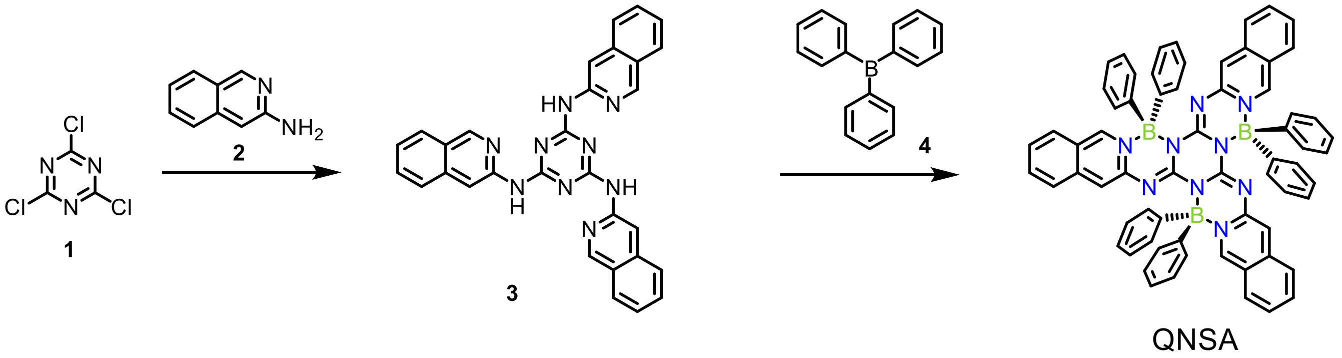

Scheme S1. Synthetic route of QNSA.

Synthesis of 3.
Excess isoquinoline-3-amine (**2**, 4.50 g, 31.3 mmol) and cyanuric chloride (**1**, 1.84 g, 10.0 mmol) were dissolved in toluene (40 mL) with *N*,*N*-diisopropylethylamine (DIPEA, 5 mL), then the mixture was refluxed under argon for 12 h. After cooling to room temperature, the solvent was removed under reduced pressure and a black solid was gained. The solid was dispersed in C_2H_5OH, filtered and the brown residue that was gained contained the target product and few by-products. The residue was dried in vacuum and used for the next reaction without further purification. The yield of the crude product is about 75%. MS (MALDI-TOF, positive mode): *m/z*: $[M]^+$ calculated for $C_{30}H_{21}N_9$: 507.1920 (100.0%), found 507.0867 $[M]^+$.

Synthesis of QNSA.

The intermediate compound **3** (0.51 g, 1 mmol) and excess triphenylborane (**4,** 1.0 g, 4.1 mmol) were dissolved in toluene (40 mL). The mixture was degassed three times and refluxed under argon for 48 h. After cooling down to room temperature, the solvent was evaporated under reduced pressure and the crude purified by column chromatography (silica, DCM:PE = 1:1 V:V). Recrystallization in the mixture of dichloromethane/ethanol gave a pure yellow powder. The yield of the target molecule in the second step is about 20% and the total yield of these whole synthetic route is about 15%. ^{13}C NMR was tested in Chloroform-*d* but the ^{1}H NMR was tested in DMSO-d_6 because the signal of the Chloroform-*d* overlapped with the signal of the QNSA at 7.26 ppm. ^{1}H NMR (400 MHz, DMSO-d_6, Figure S1) δ 8.73 (s, 3H), 8.01 (d, J = 8.6 Hz, 3H), 7.81 – 7.69 (m, 6H), 7.57 (d, J = 7.5 Hz, 12H), 7.42 (t, J = 7.5 Hz, 3H), 7.27 (t, J = 7.4 Hz, 12H), 7.16 (t, J = 7.3 Hz, 6H), 6.75 (s, 3H). ^{13}C NMR (126 MHz, Chloroform-*d,* Figure S2) δ 148.28, 148.22, 146.51, 139.93, 134.71, 133.56, 133.18, 133.00, 132.81, 131.09, 129.06, 128.96, 127.95, 127.64, 127.06, 125.79, 125.46, 125.37, 123.45, 115.08. MS (MALDI-TOF, positive mode): *m/z*: $[M]^+$ calculated for $C_{66}H_{48}B_3N_9$: 999.4312, found 999.5266 $[M]^+$.

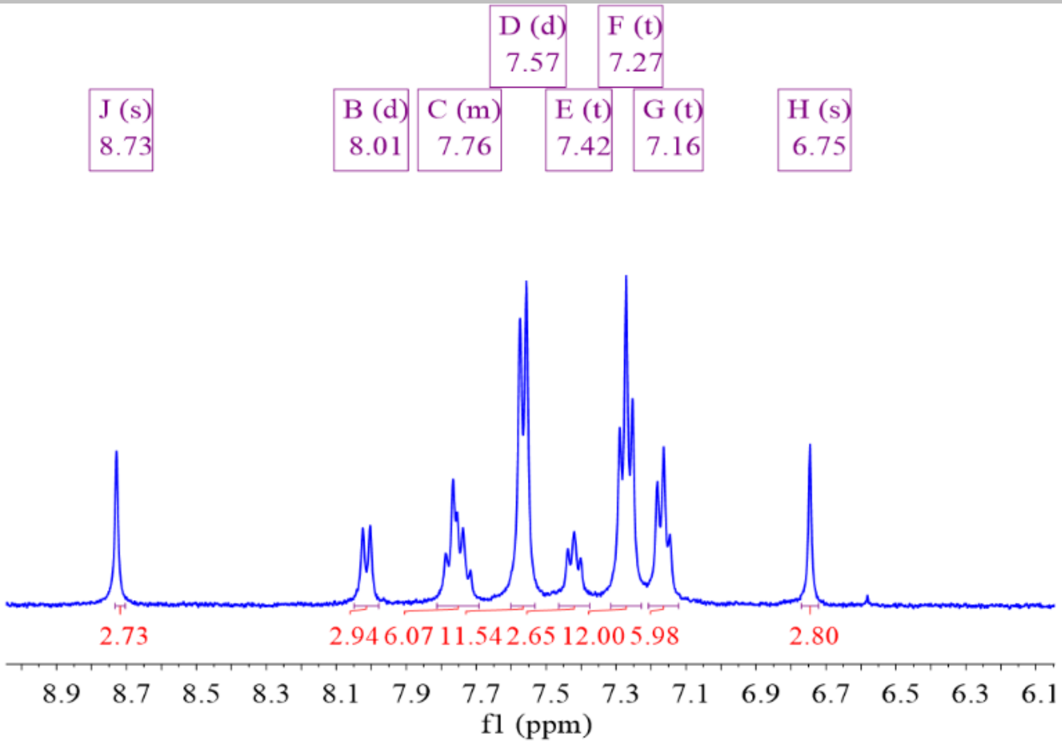

Figure S1. ^{1}H NMR spectra of QNSA, recorded at 400 MHz in DMSO-d_6 solution and room temperature.

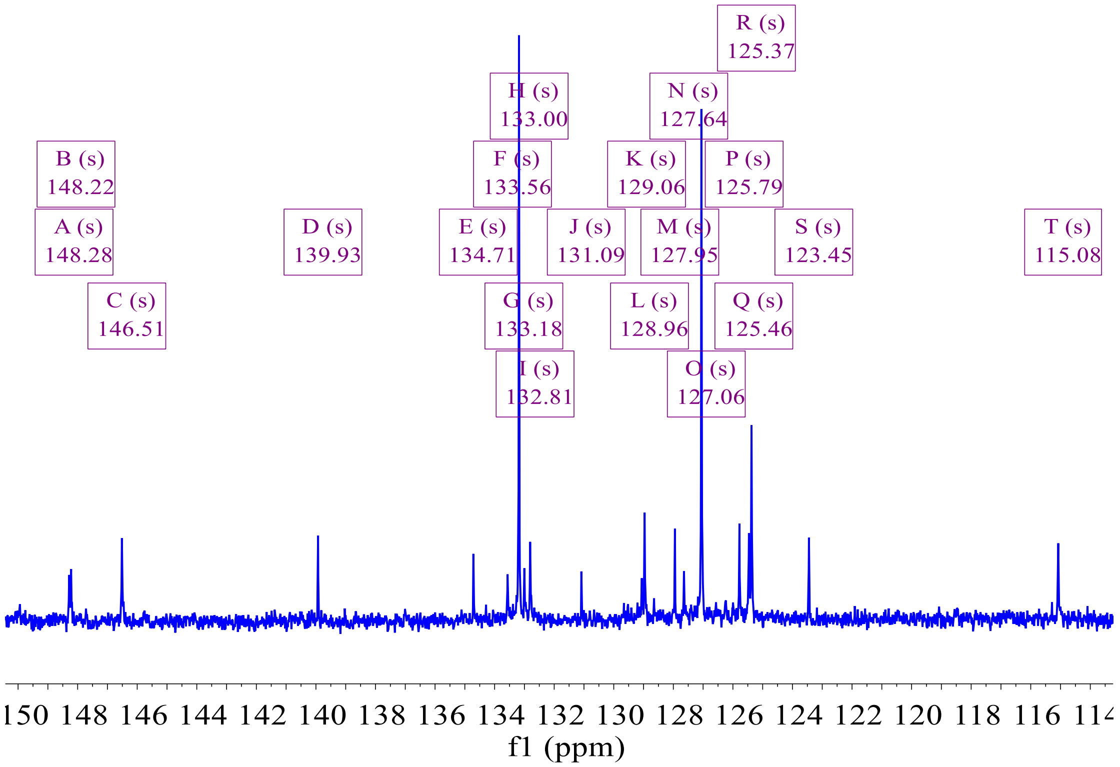

Figure S2. ^{13}C NMR spectra of QNSA, recorded at 126 MHz in chloroform-*d* solution and room temperature.

3. Thermo Gravimetric Analysis and Differential Scanning Calorimetry

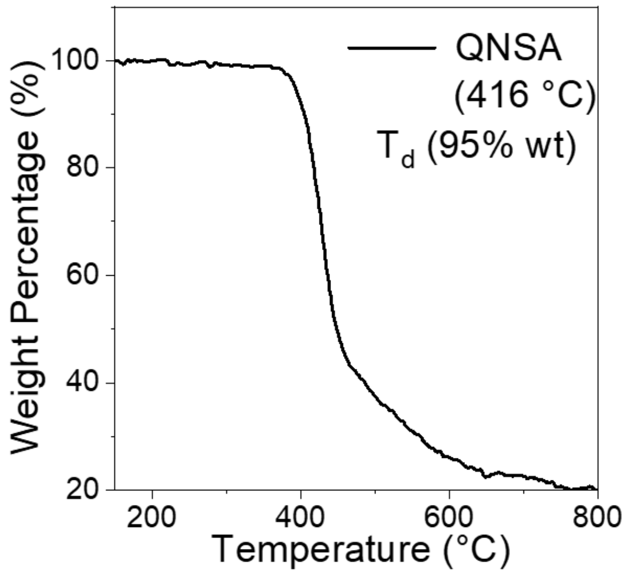

Figure S3. TGA curves of QNSA with heating rate about 20 ℃/min.

Melting points are not found under 300 ℃ in DSC measurement.

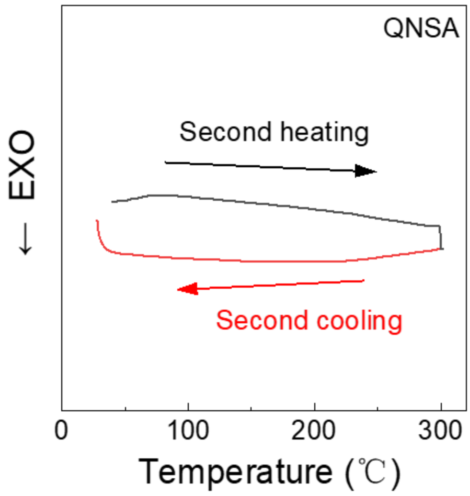

Figure S4. DSC curves of QNSA, recorded from 30 ℃ to 300 ℃ with 20 K/min heating and cooling rate. First heating and cooling were used to eliminate the thermal history.

4. Single Crystal X-ray Analysis

Table S1. Crystal data for the QNSA.

Identification code	$QNSA \cdot CH_2Cl_2$
CCDC number	2142645
Empirical formula	$C_{66}H_{48}B_3N_9 \cdot CH_2Cl_2$
Formula weight	1084.49
Temperature/K	150(2)
Crystal system	monoclinic
Space group	$P2_1/n$
a/Å	11.810(2)
b/Å	25.866(5)
c/Å	18.498(4)
α/°	90
β/°	90.23(3)
γ/°	90
Volume/$Å^3$	5651(2)
Z	4
ρ_{calcd} g/cm^3	1.275
μ/mm^{-1}	0.167
F(000)	2256.0
Crystal size/mm^3	0.08 × 0.06 × 0.05
Radiation	Cu Kα (λ = 1.54184)
2Θ range for data collection/°	2.706 to 52.542
Index ranges	$-14 \le h \le 14$, $-32 \le k \le 32$, $-22 \le l \le 14$
Reflections collected	54796
Independent reflections	11132 [R_{int} = 0.0349, R_{sigma} = 0.0225]
Data/restraints/parameters	11132/0/730
Goodness-of-fit on F^2	1.039
Final R indexes [I>=2σ (I)]	R_1 = 0.0613, wR_2 = 0.1653
Final R indexes [all data]	R_1 = 0.0649, wR_2 = 0.1684
Largest diff. peak/hole / e $Å^{-3}$	0.99/-1.55

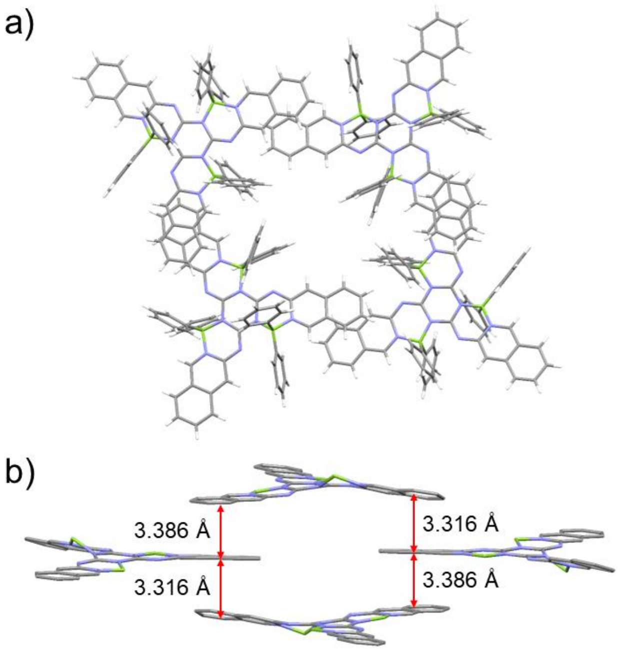

Figure S5. a) Top view and b) side view of slipped π-π-stacking arrangements involving the outer part of the π-scaffolds as isoquinoline units. The π-π stacking distances were revealed about 3.386 Å and 3.316 Å.

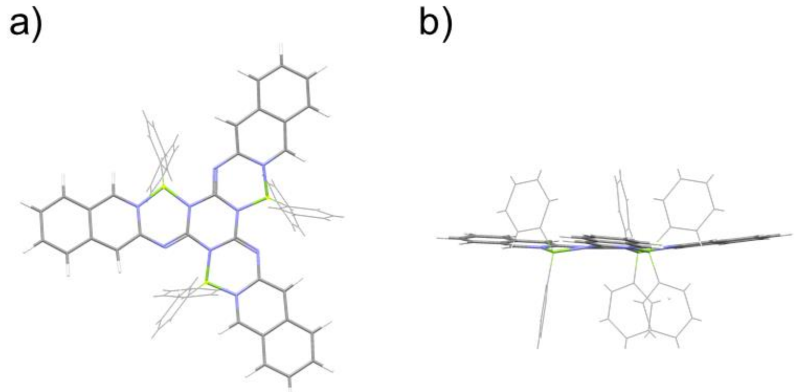

Figure S6. a) Top view and b) side view of QNSA from DFT calculation at B3LYP/6-31+G(d).

The bond lengths in QNSA are depicted in Table S2a, obtained from single crystal structures. The difference between two B-N bonds is too small to distinguish the bonding type, while the difference between C1-N1 and C2-N1 is much bigger. In addition, the bond lengths from DFT calculations, depicted in Table S2b, give the same results with that from crystal structure.

Table S2a. Bond lengths of selected bonds obtained from single crystal structure. [a]

Molecule	C1-N1 (Å)	C2-N1 (Å)	C2-N2 (Å)	B1-N2 (Å)	B1-N3 (Å)	C1-N3 (Å)	C1'-N3 (Å)
QNSA	1.311	1.354	1.341	1.610	1.616	1.361	1.380

[a] Average bond length with standard deviation of the bonds on the three arms. The bond lengths in three arms had a small difference because of the stacking.

Table S2b. Bond lengths of selected bonds obtained by DFT calculation.

Molecule	C1-N1 (Å)	C2-N1 (Å)	C2-N2 (Å)	B1-N2 (Å)	B1-N3 (Å)	C1-N3 (Å)	C1'-N3 (Å)
QNSA	1.305	1.351	1.383	1.633	1.622	1.372	1.385

For a further comparison of the bond lengths in triazine, melamine and its bond lengths obtained from crystal structure and DFT are shown in Figure S7.[3] The C-N bonds of triazine ring in melamine are shorter than C1-N3 and C1'-N3 in QNSA, and the C-N bonds out of the triazine ring in melamine are longer than the corresponding C1-N1. As a result, the triazine units in QNSA is distinguished from the triazine unit in melamine, indicating that the boron-locking mutates the structure of the central ring.

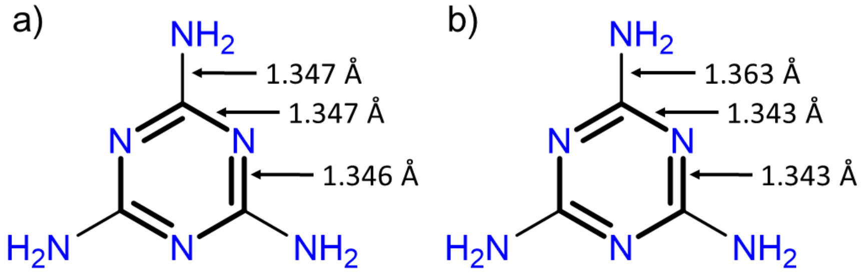

Figure S7. Melamine and its bond lengths as obtained from a) crystal structure and b) DFT calculation.

5. Resonance Structures

QNSA contains nine N. There are four possible resonance structures of QNSA backbone empirically (Figure S8). According to the bond lengths of the C-N bond in the crystal structure, the optimized configuration (DFT) and the following AICD and NICS analysis, we can conclude that **QNSA(1)** is the most relevant backbone resonance structure. Thus, it has the central non-aromatic triazine ring and outer aromatic isoquinoline units.

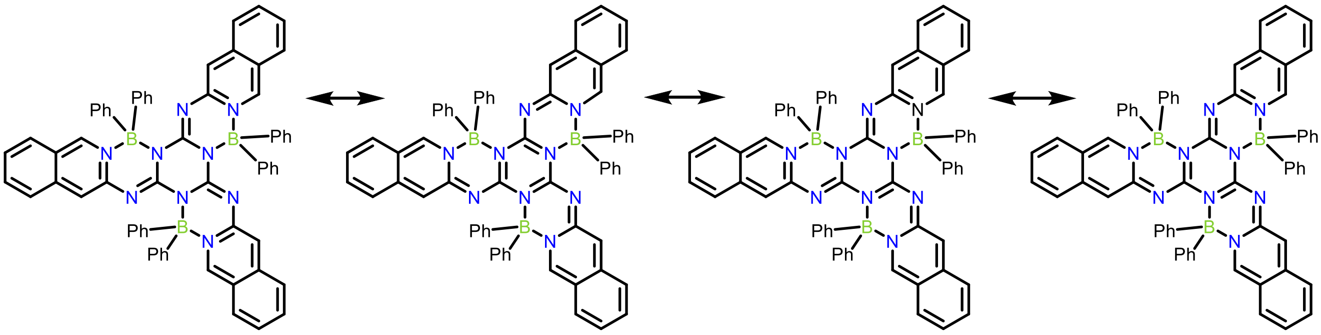

QNSA(1) **QNSA(2)** **QNSA(3)** **QNSA(4)**

Figure S8. Possible resonance structures of QNSA backbone.

6. Aromaticity

The nucleus independent chemical shift (NICS)[4] values are calculated at the B3LYP/6-31+G(d) level of theory. The value of triazine ring in melamine is negative, shown in Figure S9b, indicating this triazine ring is aromatic, different to the triazine ring in QNSA. The reduced aromaticity of the triazine ring strongly suggests that the boron-locking influence the distribution of the π-electrons.

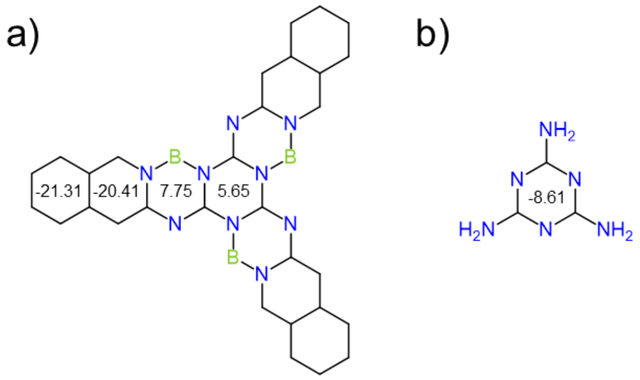

Figure S9. NICS(1)$_{zz}$ values of a) QNSA and b) melamine.

7. π-electron Analysis

The π-electronic contribution of the backbone is calculated and visualised.[5] The B atoms clearly do not contribute π-electron density. However, the N atoms form bridges to maintain the conjugation of the backbone.

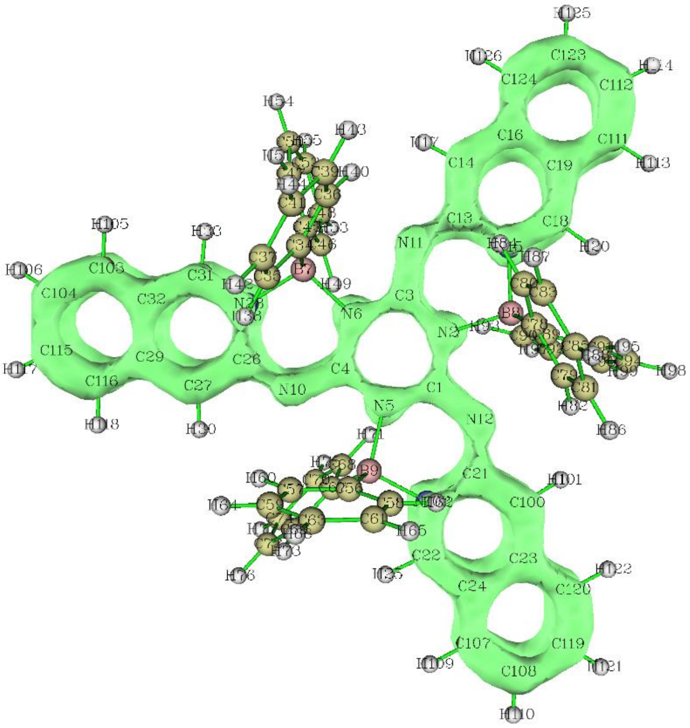

Figure S10. π-electron analysis in backbone of QNSA, the isosurface value is 0.40.

8. Electrostatic Potential Map (ESP)

Figure S11a shows the ESP map of QNSA on the vdW surface up to an electron density of 0.001 electronbohr^{-3}. The central triazine ring is blue, revealing that it is electron deficient; the outside quinoline with orange colour are electron rich. Compared with starphene (Figure S11b), the central triazine core in QNSA shows strong electronic negativity with blue colour in ESP map, displaying a great charge polarization of the π-surface.

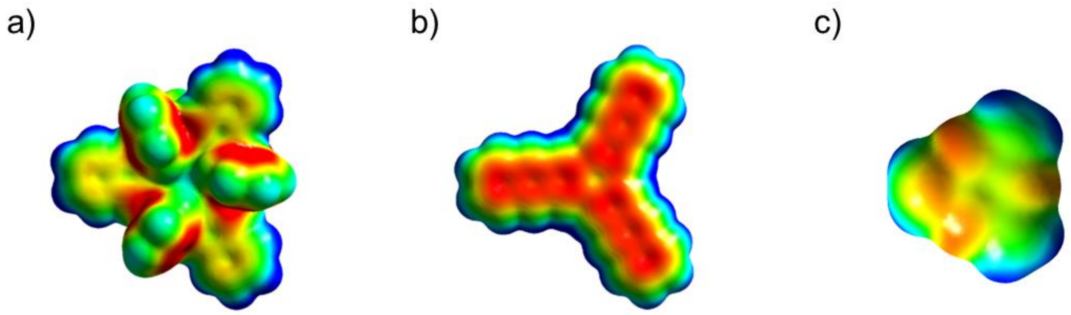

Figure S11. ESP maps of a) QNSA, b) starphene and c) melamine.

9. Absorption and Photoluminescence Spectra

The steady-state absorption and PL spectra at room temperature for QNSA were tested in various solvents (10^{-5} M solution, room temperature), as shown in Figure S12. The absorption spectra and PL spectra are insensitive to the polarity of the solvents. We also note that the PL intensity is insensitive to the polarity of the solvents.

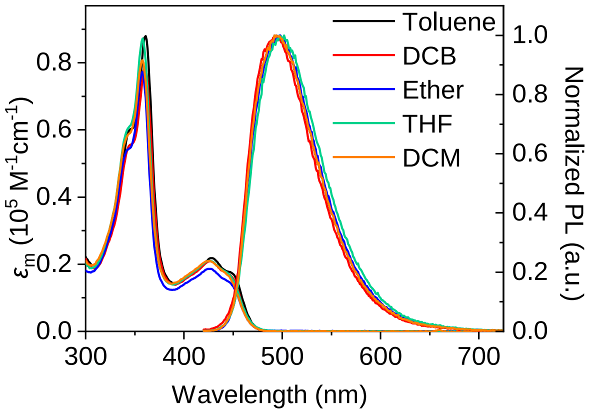

Figure S12. Absorption spectra (left) and normalized PL spectra (right) of QNSA in various solvents.

10. Frontier Orbitals

The frontier orbitals of QNSA are obtained by DFT with the level of B3WP91/def2TZVP, shown in Figure S13.

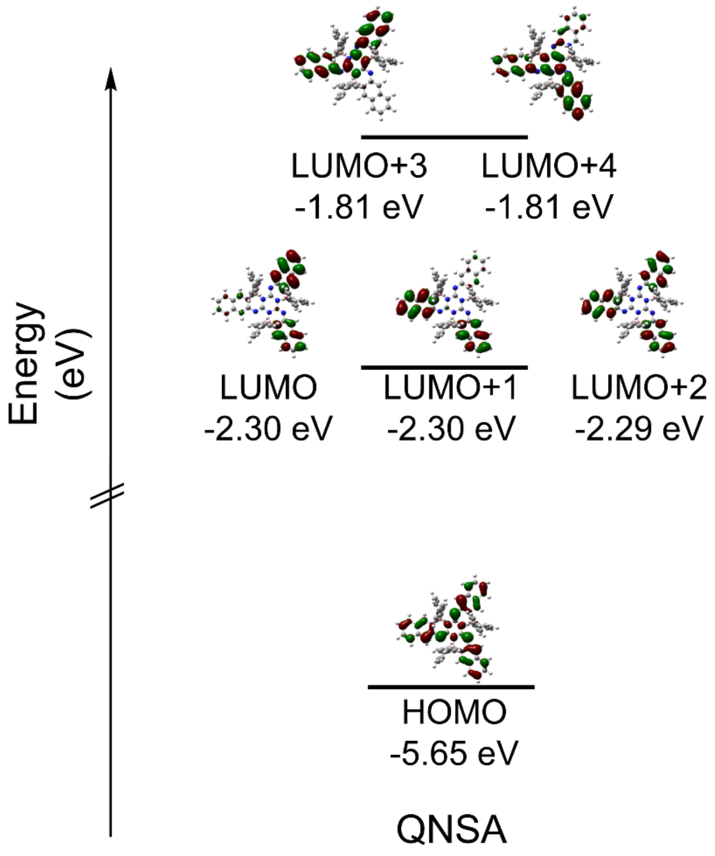

Figure S13. Frontier orbitals of QNSA.

11. Calculated Excited States and Spectra of Starphene

The calculated energy levels and oscillator strengths of starphene are listed in Table S3 and the calculated spectrum is shown in Figure S14. This molecule had degenerated energy levels, and the excited state with the highest oscillator strength is a higher excited state; the lowest transitions to the excited states are not allowed because of the symmetry-forbidden character. Additionally, in the calculated absorption spectrum, a strong absorption band resides in the ultra-violet region because the lowest excited states are symmetry-forbidden.

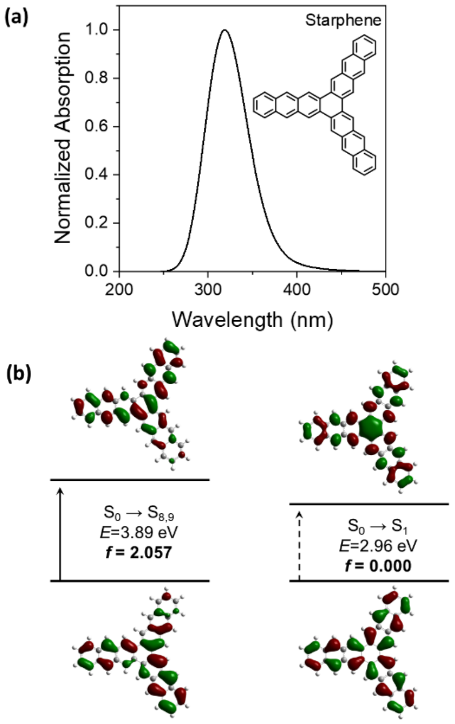

Figure S14. (a) Calculated absorption spectrum of starphene. (b) NTOs of starphene for the electronic transition from ground state to the excited states.

Table S3. Energy levels and oscillator strengths of starphene.

Transition	Energy levels (eV)	Oscillator strength
$S_0 \rightarrow S_1$	2.9562	0.0000
$S_0 \rightarrow S_{2,3}$	3.1946	0.0353
$S_0 \rightarrow S_4$	3.4167	0.0000
$S_0 \rightarrow S_{5,6}$	3.4206	0.0002
$S_0 \rightarrow S_7$	3.7812	0.0000
$S_0 \rightarrow S_{8,9}$	3.8881	2.0565
$S_0 \rightarrow S_{10}$	4.2369	0.0390

References

[1] J. E. Borger, A. W. Ehlers, M. Lutz, J. C. Slootweg, K. Lammertsma, *Angew. Chem. Int. Ed.* **2016**, *55*, 613– 617; *Angew. Chem.* **2016**, *128*, 623–627.
[2] O. V. Dolomanov, L. J. Bourhis, R. J. Gildea, J. A. K. Howard, H. Puschmann, *J. Appl. Crystallogr.* **2009**, *42*, 339– 341.
[3] A. Cousson, B. Nicolaï, F. Fillaux, *Acta Crystallogr. Sect. E* **2005**, *61*, 222-224.
[4] Z. Chen, C. S. Wannere, C. Corminboeuf, R. Puchta, P. V. R.Schleyer, *Chem. Rev.* **2005**, *105*, 3842 – 3888.
[5] T. Lu, Q. Chen, *Theor. Chem. Acc.* **2020**, *139*, 1-12.
